# Regulatory Manner and Role of Circular RNAs in the Microsporidian Invasion of Asian Honeybee

**DOI:** 10.3390/ani16162451

**Published:** 2026-08-07

**Authors:** Xue Yang, Jianpeng Lei, Yuwei Zhang, Jiashuo Zhu, Xueqin Li, Jianfeng Qiu, Dafu Chen, Rui Guo

**Affiliations:** 1 College of Bee Science, Fujian Agriculture and Forestry University, Fuzhou 350002, China; y2745860171@163.com (X.Y.); 18859317726@163.com (J.L.); 13759839701@163.com (Y.Z.); 15032998960@163.com (J.Z.); 18750773896@163.com (X.L.); jfqiu@fafu.edu.cn (J.Q.); 2 National & Local United Engineering Laboratory of Natural Biotoxin, Fuzhou 350002, China; 3 Apitherapy Research Institute, Fujian Agriculture and Forestry University, Fuzhou 350002, China

**Keywords:** *Vairimorpha ceranae*, *Apis cerana cerana*, circular RNA, ceRNA, energy metabolism

## Abstract

*Vairimorpha ceranae*, an obligate intracellular parasitic fungus, infects the midgut of worker *Apis cerana cerana*, disrupts nutritional metabolism and immune homeostasis of hosts, and poses severe threats to honeybee health and the sustainable development of apiculture. To date, the expression dynamics of circular RNAs (circRNAs) during pathogen infection and their regulatory functions as competing endogenous RNAs (ceRNAs) remain poorly characterized. In this study, circRNA sequencing was performed on purified *V. ceranae* spores (VcCK), as well as the midguts of *A. cerana cerana* worker bees at 7 days post-infection (VcT1) and 10 days post-infection (VcT2). A total of 8,986,470 and 315 circRNAs were identified from the three groups, with their lengths predominantly ranging from 200 to 600 nt. Differential expression analysis screened 575 and 594 differentially expressed circRNAs (DEcircRNAs) from the VcCK vs. VcT1 and VcCK vs. VcT2 comparison groups, respectively. The host genes generating these DEcircRNAs were annotated to 316 and 310 GO terms, alongside 167 KEGG pathways. CeRNA network analysis revealed that DEcircRNAs including novel_circ_006653 target multiple miRNAs, and their downstream target mRNAs are significantly enriched in energy metabolism pathways such as carbon metabolism and glycolysis/gluconeogenesis. RT-qPCR validation exhibited high consistency with transcriptome sequencing data. Further analyses demonstrated that *V. ceranae* infection drastically remodels the circRNA expression landscape: the total number of circRNAs was sharply reduced, and the dominant circRNA subtype shifted from antisense circRNAs to single-exon circRNAs. Several DEcircRNAs (novel_circ_006653, novel_circ_004839, novel_circ_005596, etc.) may participate in pathogen infection progression via the ceRNA regulatory axis by modulating host energy metabolism, immune responses and cellular biological processes. This study provides novel insights into the molecular interaction mechanisms between microsporidian parasites and their honeybee hosts, and identifies potential molecular targets for the prevention and control of microsporidiosis.

## Simple Summary

*Vairimorpha ceranae*, a microsporidian fungus that specifically parasitizes the honeybee midgut, impairs nutrient absorption and immune function of bees, thereby posing severe threats to the health of *Apis cerana cerana*. Circular RNAs (circRNAs) are a class of structurally stable, degradation-resistant non-coding RNAs. Nevertheless, their expression patterns and biological roles during *V. ceranae* infection in honeybees remain largely elusive. In the present study, high-throughput circRNA sequencing was conducted to comparatively characterize circRNA profiles of purified pathogen spores (uninfected control), as well as microsporidia isolated from honeybee midguts at 7 and 10 days post-infection. The results revealed a dramatic reduction in the total number of microsporidian circRNAs post infection, accompanied by a shift in the dominant circRNA subtype from antisense circRNAs to single-exon circRNAs. Hundreds of differentially expressed circRNAs were identified. CeRNA network prediction indicated that these circRNAs function as endogenous miRNA sponges to sequester microRNAs (miRNAs), indirectly modulating the transcriptional activity of downstream genes associated with energy metabolism and immunity. RT-qPCR validation corroborated the reliability of sequencing data. Key circRNAs including novel_circ_006653 may facilitate the survival and proliferation of pathogens within host bees by regulating host energy supply and immune responses. This study systematically uncovers the dynamic expression patterns and latent regulatory functions of microsporidian circRNAs during host infection for the first time, offering novel molecular targets and theoretical foundations for the prevention and treatment of honeybee microsporidiosis.

## 1. Introduction

Bees are indispensable pollinating insects in nature, playing an irreplaceable role in maintaining ecological balance, ensuring food security, and preserving biodiversity [1,2]. The *Apis cerana cerana* possesses advantages such as strong foraging ability, efficient utilization of scattered nectar sources, keen olfaction, agile flight, and strong tolerance to cold and resistance to diseases [3,4]. However, under the intensification of intensive farming and environmental stress, pathogen infection has become a key factor threatening the health of *A. c. cerana* and is causing colony decline.

*Vairimorpha ceranae* is an obligate intracellular parasitic fungus. Its spores exist in a dormant state outside host cells, maintaining only basic life activities [5,6,7]. Honeybees ingest *V. ceranae* spores through contaminated water or food. The spores are activated and germinate in the weakly alkaline environment of the midgut, where increased internal osmotic pressure prompts the rapid eversion of the polar filament, injecting the sporoplasm into host cells [8,9]. This infection leads to nutritional metabolic imbalance, immune dysfunction, and reduced lifespan in honeybees, severely endangering colony health and apiculture production [10]. Fan et al. [11] comprehensively revealed the extensive negative impacts of *V. ceranae* infection on energy metabolism, immune defense, cell structure, and lifespan of *A. c. cerana* workers, providing new insights into the interaction between Asian honeybees and microsporidia. Currently, there is a lack of effective control measures for microsporidiosis, and the core bottleneck lies in the incomplete elucidation of the molecular mechanisms underlying pathogen infection and host–pathogen interactions.

Circular RNAs (circRNAs) are a class of endogenous non-coding RNA (ncRNAs) molecules ubiquitously present in eukaryotic cells. They are characterized by high stability and strong expression specificity, and lack protein-coding capacity [12]. Compared with model organisms such as humans, research on circRNAs in fungi has been mainly limited to several model species, including *Ascosphaera apis* [13], *Cryptococcus neoformans* [14], and *Ganoderma lucidum* [15]. Nevertheless, the widespread adoption of high-throughput sequencing technologies in recent years has facilitated substantial advances in the identification of fungal circular RNAs (circRNAs). Guo et al. [16] pioneered the genome-wide identification of circRNAs in *V. ceranae*. Zhou et al. [17] identified 8848 circRNAs in *Magnaporthe oryzae* and revealed their differential enrichment between mycelial and spore stages. Hu et al. [18] reported that the functional circRNA circDS-1 regulates dimorphic switching and virulence in the human pathogenic fungus *Talaromyces marneffei*. Ubiquitously distributed across fungi and endowed with regulatory functional potential, circRNAs have progressively emerged as a novel frontier in fungal biological research.

In the last decade, transcriptomic study on *V. ceranae* has yielded pivotal breakthroughs. Gen et al. [19] utilized transcriptome sequencing to profile differentially expressed genes during the *V. ceranae* infection of *Apis mellifera ligustica*, identifying virulence factor-encoding genes responsible for spore wall proteins, polar tube proteins, and ricin B lectins. Li et al. [20] and Chen et al. [21] independently deciphered the dynamic transcriptomic landscapes of this microsporidian following infection of *Apis mellifera* and *Apis cerana*, respectively. Fan et al. performed comparative transcriptome analysis of *V. ceranae* during the infection of *A. c. cerana* workers, detecting the activation of most of the virulence factor genes [22]. By employing strand-specific cDNA library-based RNA sequencing technology, Guo et al. discovered 83 lncRNAs in *V. ceranae* spores [23], offering valuable resources for deep investigation of their regulatory functions as well as underlying mechanisms. These findings collectively lay a foundation for dissecting the infection mechanisms of this pathogen and the underlying pathogen–host interaction networks.

In this study, deep sequencing was performed on purified spores of *V. ceranae* (VcCK) and the midgut tissues of *A. c. cerana* workers at 7 days (VcT1) and 10 days (VcT2) post-infection. Based on the high-quality data obtained, we systematically identified and analyzed circRNAs of *V. ceranae* using bioinformatics approaches, screened DEcircRNAs of *V. ceranae* after infecting the host, and analyzed their regulatory functions. Furthermore, we predicted and analyzed the protein-coding potential of DEcircRNAs, and molecularly validated the back-splicing junction sites and expression trends of DEcircRNAs. This study aims to deeply dissect the infection mechanism of *V. ceranae* at the omics level and provide potential molecular targets for the control of *V. ceranae* disease.

## 2. Materials and Methods

### 2.1. Purification of the V. ceranae Strain

According to the method described by Guo et al., the *V. ceranae* was administered to the bees in the laboratory, and the *V. ceranae* spores were purified [16,24]. Dissect the midguts of *A. c. cerana* workers infected with *V. ceranae* spores, wash with phosphate-buffered saline (PBS, Yeasen Biotechnology (Shanghai) Co., Ltd., Shanghai, China), and then homogenize and filter. Centrifuge the filtrate at 845 rpm for 15 min. Resuspend the pellet in sterile water and centrifuge at 2348 rpm for 15 min; repeat this wash twice. Then, resuspend the pelleted spores in 25% Percoll and layer them onto a pre-formed Percoll density gradient (100%, 75%, 50%, and 25%). Centrifuge the gradient at 12,210 rpm for 30 min. Carefully aspirate the milky-white spore band to obtain purified spores (control group, VcCK).

### 2.2. Inoculation of V. ceranae Spores in Workers

*A. c. cerana* workers were collected from three colonies. The preparation of midgut samples from *Apis cerana* worker bees followed the previously established rearing protocol [25]. Inoculation of *A. c. cerana* workers and preparation of midgut were performed according to previous protocols [26]. Briefly, sealed brood frames containing pupae close to emergence were placed in an incubator at 34.5 ± 0.5 °C with 70% relative humidity for 12 h. Newly emerged workers were transferred into sterile plastic boxes (with holes punched on the sides for ventilation) at a density of 30 bees per cage. A feeder containing 50% (*w/v*) sterile sucrose solution was placed on top of each cage. Bees immediately after emergence were designated as 0 h, and after 24 h of further incubation, they were subjected to a 2 h starvation period. Bees in the treatment group were each fed 5 μL of sucrose solution containing 1 × 10^6^ spores of *Vairimorpha ceranae*. Three biological replicates were set up for the experiment. At 7 days post-inoculation (7 dpi) and 10 dpi, the midgut tissues were dissected. Every three midguts were placed into one RNA-Free centrifuge tube. The 7 dpi treatment group was designated as VcT1, and the 10 dpi treatment group was designated as VcT2. The remaining samples were stored at −80 °C in an ultra-low temperature freezer for subsequent use.

### 2.3. circRNA Sequencing

TRIzol™ Reagent (Thermo Fisher Scientific, Waltham, MA, USA) was adopted to isolate total RNA from *V. ceranae* spores (VcCK) and *V. ceranae*-infected bee midguts (VcT1 and VcT2). After eliminating ribosomal RNA contaminants, RNase R treatment was implemented to degrade linear RNA molecules within the RNA extracts. The circular RNA pool obtained after enrichment was fragmented into short fragments, followed by a reverse transcription reaction primed with random hexamer primers. Double-stranded complementary DNA (cDNA) was further synthesized and purified, and sequencing libraries were subsequently built-in strict accordance with standard Illumina library preparation workflows. The final constructed cDNA libraries were subjected to high-throughput sequencing on the Illumina HiSeq™ 2500 sequencing platform, with sequencing services completed by Guangzhou GeneDeer Biotechnology Co., Ltd. (Guangzhou, China).

### 2.4. Quality Control and Filtration of Sequencing Data

Raw sequencing data of the VcCK, VcT1 and VcT2 groups were processed to obtain high-quality (HQ) clean reads. To completely eliminate interference from host-derived sequences and exclusively retain reads originating from *V. ceranae*, a two-step genome alignment filtering strategy was performed: all HQ clean reads were first mapped to the reference genome of *A. c. cerana*. All reads with successful alignment to the host genome were discarded, and only unmapped anchor reads were retained for subsequent parasite genome alignment. The reserved unmapped reads were extracted, and the 20 bp terminal sequences of each read were intercepted to generate anchor reads. These anchor reads were realigned to the *V. ceranae* reference genome (GCF_000182985.1). The final alignment files were imported into Find-circ (Version 1.1) software for genome-wide identification of parasite-specific circular RNAs.

### 2.5. miRNA Omics Data

High-quality sRNA-seq data were obtained in earlier study from both *V. ceranae* spores and the midguts of *A. c. cerana* at 7 d and 10 d post-infection, as detailed in reference [27].

### 2.6. Analysis of DEcircRNAs and Their Source Genes

To quantify circRNA abundance, we adopted the RPM approach, defined as the number of back-splicing junction reads per million total mapped reads. The abundance values were then log-transformed using the formula lg (RPM + 1) for all samples, including the VcCK, VcT1, and VcT2. Differential expression analysis between pairwise groups (VcCK vs. VcT1 and VcCK vs. VcT2) was carried out utilizing the DESeq package [22], with statistically significant thresholds set at an absolute |log2 (Fold change) | ≥ 1 and *p* ≤ 0.05. It is known that circRNAs may modulate the transcriptional activity of their parent genes through interactions with RNA polymerase II, U1 small nuclear ribonucleoprotein, and promoter regions. To determine the genomic origins of the identified DEcircRNAs, we mapped the anchor reads derived from both termini of each circRNA to the reference genome of *V. ceranae* (assembly ASM98816v1) with the alignment tool Bowtie [28]. A circRNA was assigned to a specific host gene only when both of its terminal reads aligned to exonic regions of the same gene. Subsequently, the resulting parental genes were submitted to the GO (http://www.geneontology.org/) (accessed on 9 December 2025) and KEGG (http://www.geneontology.org/) (accessed on 9 December 2025) databases for functional annotation and pathway assignment, respectively.

### 2.7. Targeting Relationship Prediction and Regulatory Network Analysis

Using the miRNA-omics datasets acquired in prior analyses, we screened miRNAs that may be bound and regulated by differentially expressed circRNAs (DEcircRNAs), so as to map regulatory associations between DEcircRNAs and their target miRNAs. Afterwards, target mRNAs corresponding to these microRNAs were predicted, and a regulatory network of DEcircRNA-miRNA-mRNA was built on this basis. Cytoscape version 3.2.1 was adopted for network visualization. GO functional annotation and KEGG pathway enrichment analyses were finally carried out for the obtained target mRNAs.

### 2.8. PCR Validation

Divergent primers targeting the back-splicing junction sites were designed with Primer Premier 5 (Table 1), using the nucleotide sequences of the circRNAs as a reference. Total RNA was isolated from the VcCK, VcT1, and VcT2 samples using an RNA extraction kit (Promega, Madison, WI, USA) and served as the starting template for reverse transcription. To enrich circular RNAs, the RNA samples were digested with 3 U/µg RNase R to degrade linear RNAs. After incubation at 37 °C for 15 min, the treated RNA was then reverse-transcribed with random hexamers, and the synthesized cDNA served as the PCR template. PCR mixtures and thermal cycling were prepared according to Geng et al. [29]. The amplified products were separated by 1.5% agarose gel electrophoresis, visualized under UV light, and photographed.

### 2.9. RT-qPCR Validation

RT-qPCR reaction mixtures and thermal cycling parameters were configured according to the method of Geng et al. [29], with the actin gene (GenBank accession AB023025.1) as the endogenous control.

### 2.10. Statistical Analysis

The expression level of each circRNA was normalized by RPM (mapped back-splicing junction reads per million mapped reads). DESeq (Version 1.18.0) software was used to screen differentially expressed circRNAs with thresholds of |log_2_ (Fold change) | ≥ 1 and *p* ≤ 0.05. GraphPad Prism 8 (San Diego, CA, USA) was used to generate graphs and perform statistical analyses. RT-qPCR results are shown as the mean ± SD. Two-way ANOVA was applied to determine the statistical significance of the data. Differences were considered statistically significant at *p* < 0.05. Statistical significance was defined as ns (*p* > 0.05), * (*p* < 0.05), ** (*p* < 0.01), *** (*p* < 0.001), **** (*p* < 0.0001).

## 3. Results

### 3.1. Identification and Analysis of V. ceranae circRNAs

**Figure 1:**
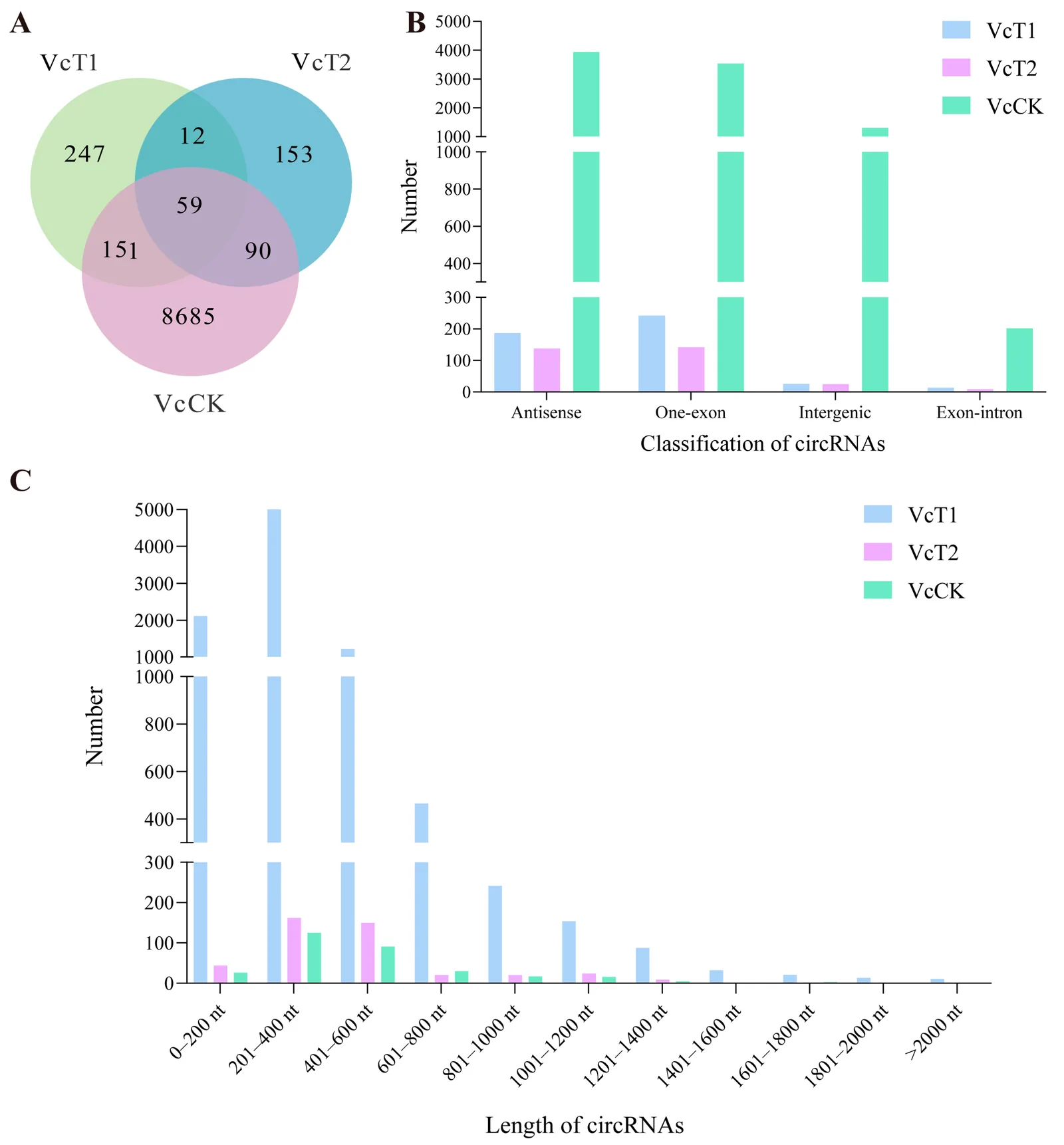
Differential analysis of circRNAs in *V. ceranae* infecting the *A. c. cerana* workers. (**A**) Venn diagram of DEcircRNAs. (**B**) Category of circRNAs. (**C**) Length distribution of circRNAs. VcCK: *V. ceranae* spores, VcT1 and VcT2: *V. ceranae* in the workers’ midguts at 7 days and 10 days post inoculation with spores.

In the VcCK, VcT1, and VcT2 groups, 8,986,470, and 315 circRNAs were predicted, respectively. Venn analysis showed that 59 circRNAs were shared among the three groups, while 8,685,247, and 153 circRNAs were unique to the VcCK, VcT1, and VcT2 groups, respectively (Figure 1A). The predominant circRNA types in the VcCK, VcT1, and VcT2 groups were antisense circRNAs, single-exon circRNAs, and single-exon circRNAs, respectively (Figure 1B). As shown in Figure 1C, the length distribution of total circRNAs was mainly concentrated between 200 nt and 600 nt.

### 3.2. Differential Expression Profile of circRNAs in V. ceranae

**Figure 2:**
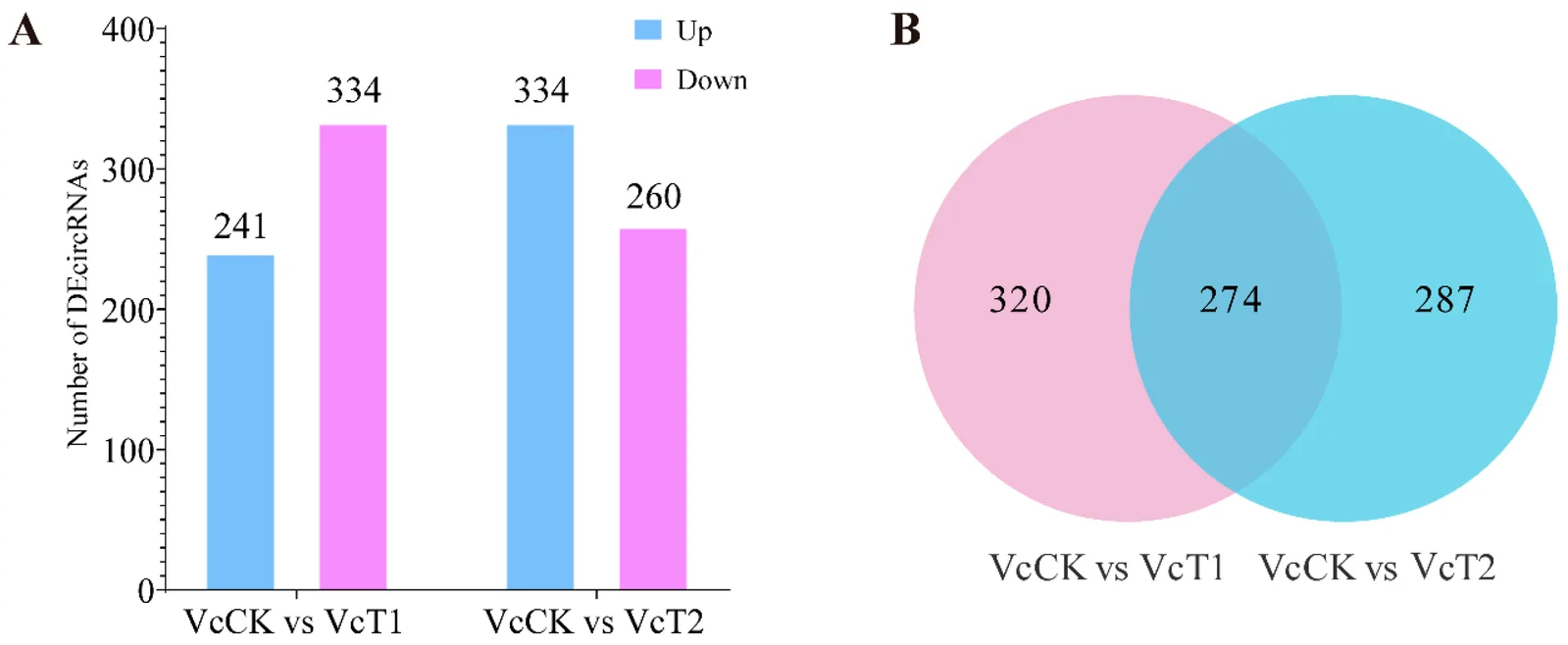
Profiling of DEcircRNAs during *V. ceranae* infection in *A. c. cerana* workers. (**A**) Up-regulated/down-regulated DEcircRNAs in two comparisons; (**B**) Venn diagram of DEcircRNAs shared between the two comparisons.

A total of 575 DEcircRNAs were screened in the VcCK vs. VcT1 comparison group, including 241 up- and 334 down-regulated circRNAs. The top three most up-regulated circRNAs were novel_circ_002688, novel_circ_002679, and novel_circ_002682, while the top three most down-regulated ones were novel_circ_005596, novel_circ_005553, and novel_circ_005597 (Table 2). In the VcCK vs. VcT2 comparison group, 594 DEcircRNAs including 334 up-regulated and 260 down-regulated circRNAs, were identified (Figure 2A), including novel_circ_002398, novel_circ_009047, and novel_circ_003865. The top three most down-regulated ones were novel_circ_005596, novel_circ_005553, and novel_circ_005597 (Table 3). Further analysis showed that 274 DEcircRNAs were shared between these two comparison groups, while 320 and 287 DEcircRNAs were specific to each comparison group, respectively (Figure 2B).

### 3.3. Functional Annotation and Pathway Enrichment of Source Genes of DEcircRNAs

**Figure 3:**
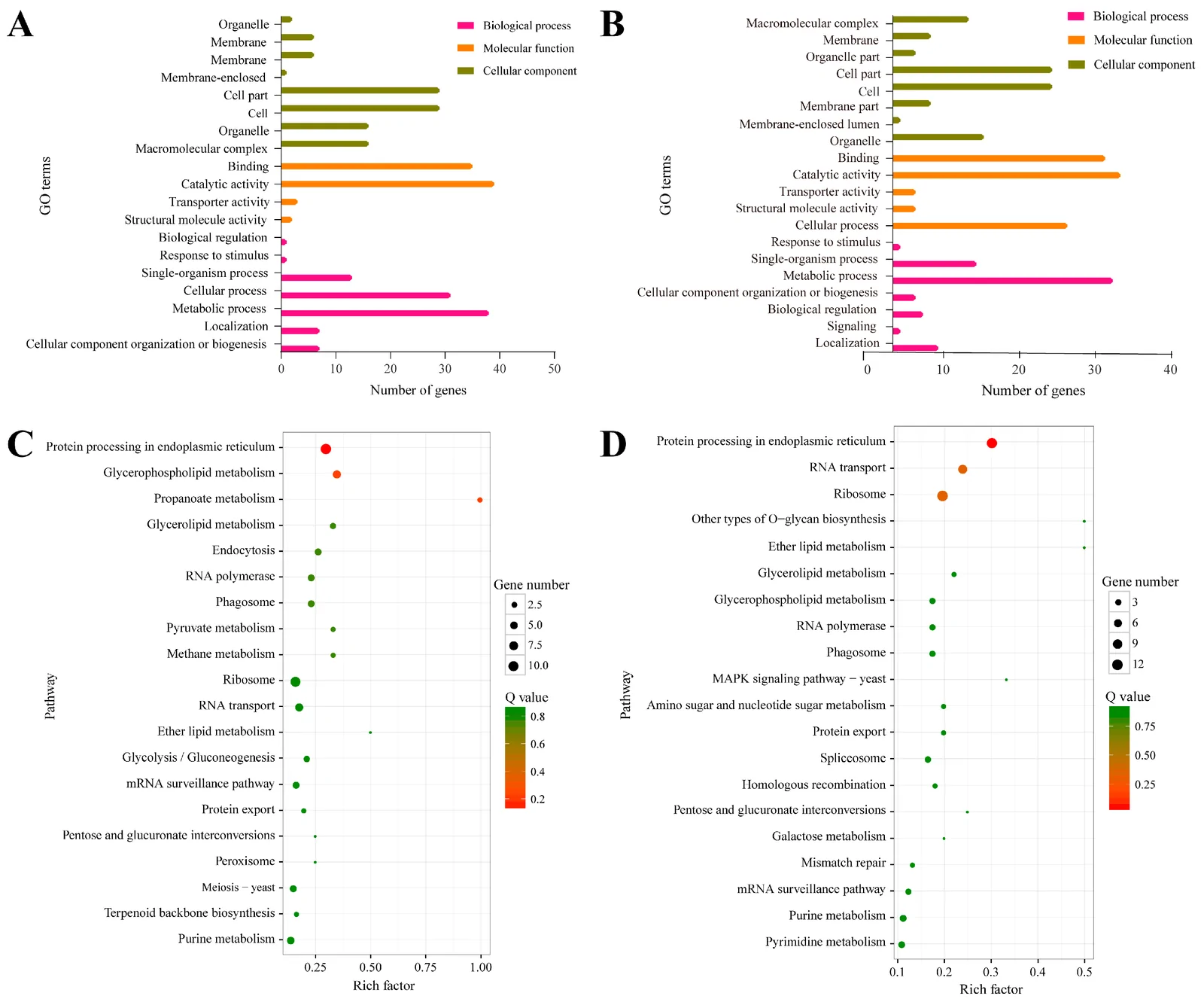
GO terms and KEGG pathways of parental genes of DEcircRNAs from *V. ceranae* infecting *A. c. cerana* workers. (**A**) Circle plot of GO terms of parental genes of DEcircRNAs in the midguts at 7 days post-infection (dpi). (**B**) Circle plot of GO terms of parental genes of DEcircRNAs in the midguts at 10 dpi. (**C**) Circle plot of KEGG pathways enriched by parental genes of DEcircRNAs in the midguts at 7 dpi. (**D**) Circle plot of KEGG pathways enriched by parental genes of DEcircRNAs in the midguts at 10 dpi.

In the VcCK vs. VcT1 comparison group, the source genes of DEcircRNAs were involved in three major GO categories: biological process, molecular function, and cellular component. The most abundantly annotated terms included metabolic process, cellular process, catalytic activity, binding, and cell part (Figure 3A). In the VcCK vs. VcT2 comparison group, the parental genes of DEcircRNAs were also involved in three major GO categories, with metabolic process, catalytic activity, binding, cellular process, and cell part being the most prominent (Figure 3B). Furthermore, in the VcCK vs. VcT1 comparison group, the parental genes of DEcircRNAs were associated with multiple pathways, including protein processing in the endoplasmic reticulum, glycerophospholipid metabolism, and endocytosis (Figure 3C). In the VcCK vs. VcT2 comparison group, the parental genes of DEcircRNAs were associated with more pathways, such as protein processing in the endoplasmic reticulum, ribosome, RNA transport, and the MAPK signaling pathway–yeast (Figure 3D).

### 3.4. Prediction of Target miRNAs of DEcircRNAs and Regulatory Network Analysis

**Figure 4:**
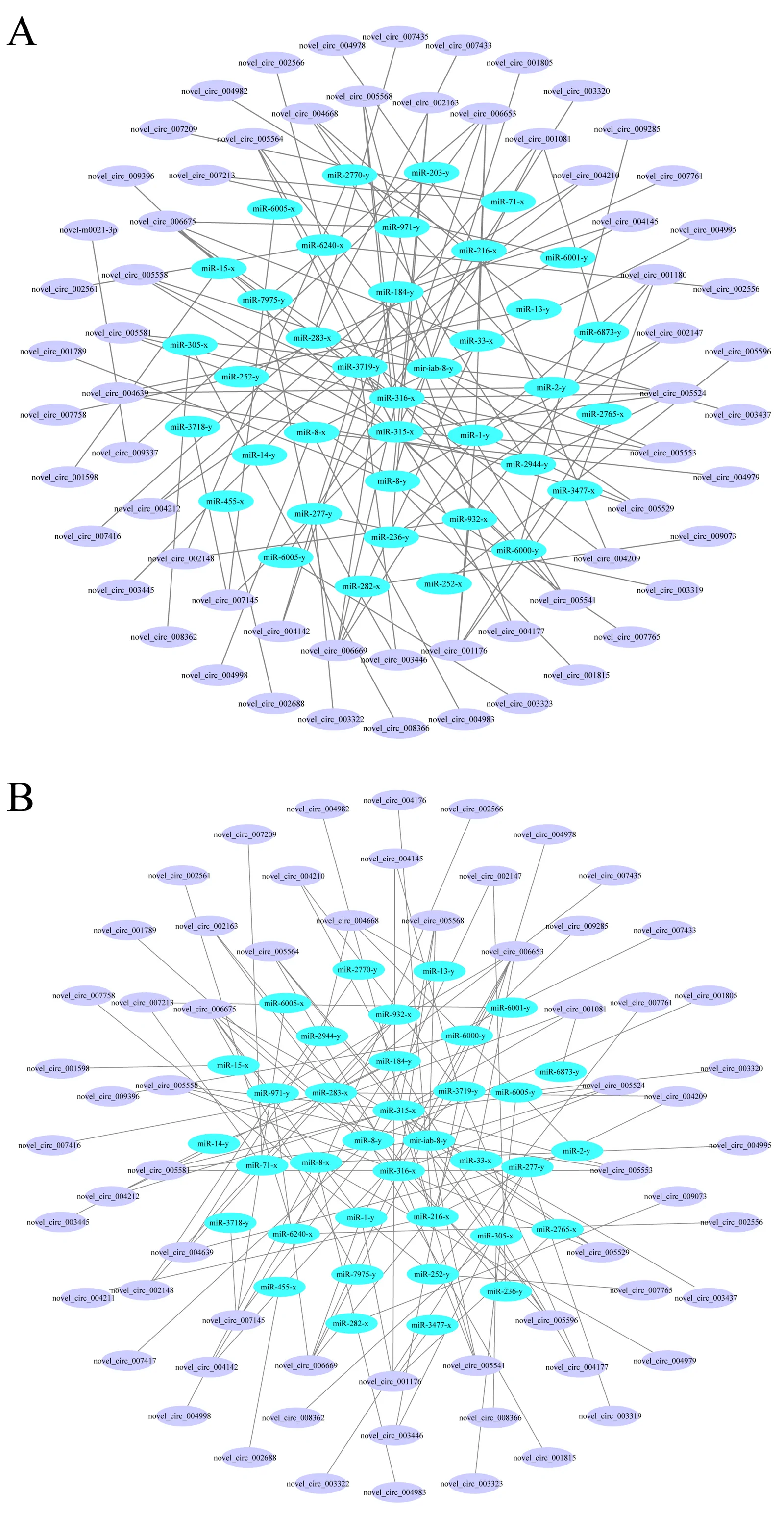
Differentially expressed circRNA–miRNA regulatory networks during *V. ceranae* infection of *A. c. cerana* workers. (**A**) circRNA–miRNA regulatory network in the VcCK vs. VcT1 comparison group; (**B**) circRNA–miRNA regulatory network in the VcCK vs. VcT2 comparison group. Purple and blue ellipses denote DEcircRNAs and DEmiRNAs, respectively. The lines represent their predicted targeting regulatory interactions.

In the VcCK vs. VcT1 comparison group, 62 DEcircRNAs were predicted to target and bind to 81 miRNAs. Among these, novel_circ_006653, novel_circ_006675, and novel_circ_006669 each targeted five miRNAs, representing the highest number of target miRNAs (Figure 4A). In the VcCK vs. VcT2 comparison group, 64 DEcircRNAs were predicted to target and bind to 77 miRNAs, with novel_circ_006653, novel_circ_006675, and novel_circ_006669 also each targeting five miRNAs (Figure 4B).

### 3.5. Functional Annotation and Pathway Enrichment of Target mRNAs

**Figure 5:**
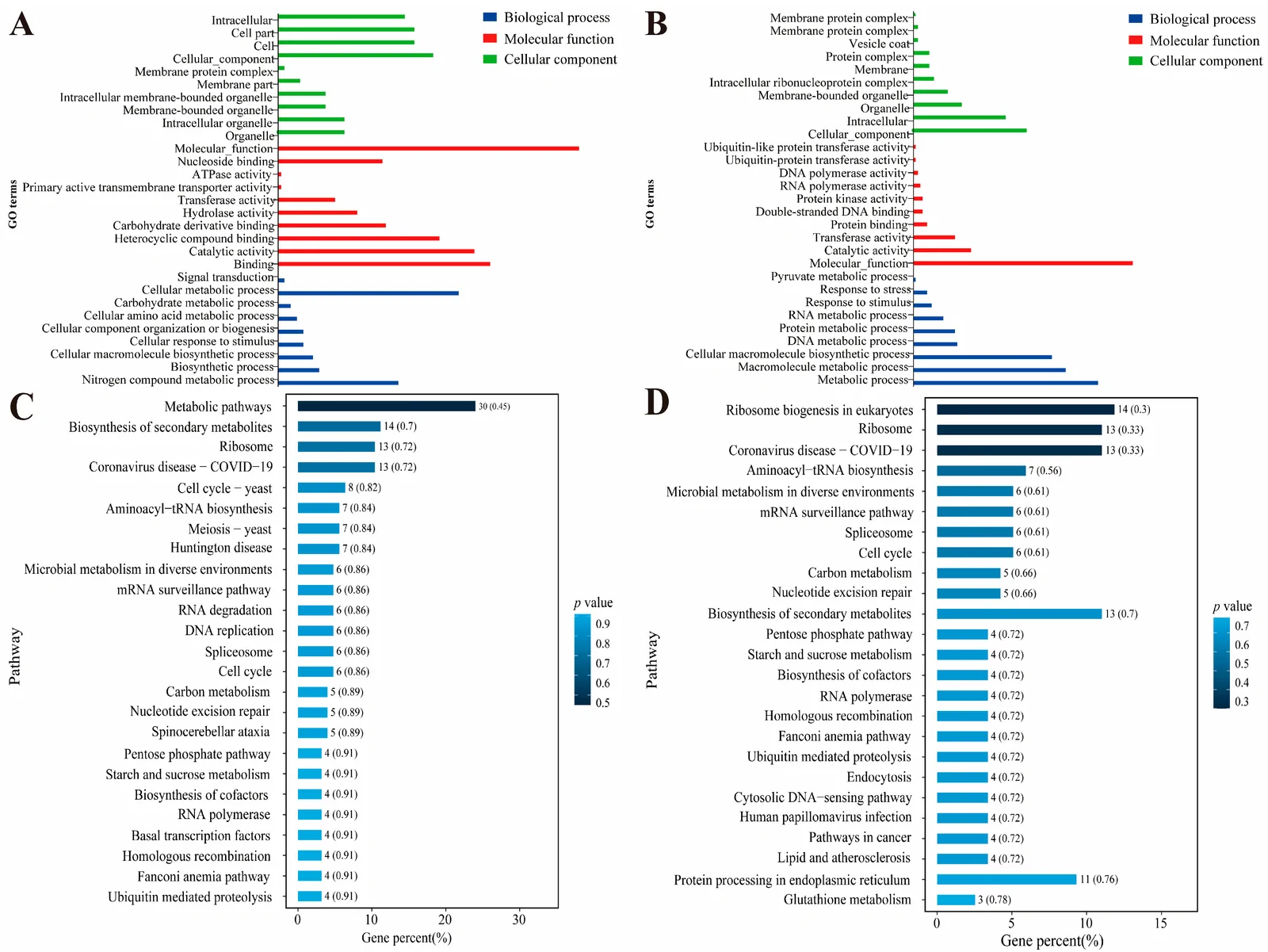
GO terms and KEGG pathways of target mRNAs regulated by DEcircRNA–miRNA interactions during *V. ceranae* infection of *A. c. cerana* workers. (**A**) GO annotation of target mRNAs in the midguts at 7 days post-infection. (**B**) GO annotation of target mRNAs in the midguts at 10 days post-infection. (**C**) KEGG pathway annotation of target mRNAs in the midguts at 7 days post-infection. (**D**) KEGG pathway annotation of target mRNAs in the midguts at 10 days post-infection.

In the VcCK vs. VcT1 comparison group, target mRNAs were annotated to 316 GO terms, including cellular component (49), molecular function (95), biological process (96), binding (67), and cell part (43) (Figure 5A). In the VcCK vs. VcT2 comparison group, target mRNAs were annotated to 310 GO terms, including cellular component (49), molecular function (95), biological process (96), binding (67), and cell part (43) (Figure 5B). Furthermore, target mRNAs in the VcCK vs. VcT1 comparison group were annotated to 176 pathways (Figure 5C), such as metabolic pathways, biosynthesis of secondary metabolites, and ribosome biogenesis. In contrast, target mRNAs in the VcCK vs. VcT2 comparison group were annotated to 129 pathways (Figure 5D), including ribosome biogenesis in eukaryotes, ribosome, and aminoacyl-tRNA biosynthesis.

### 3.6. Validation of Back-Splicing Junction Sites and Expression Trends of DEcircRNAs

**Figure 6:**
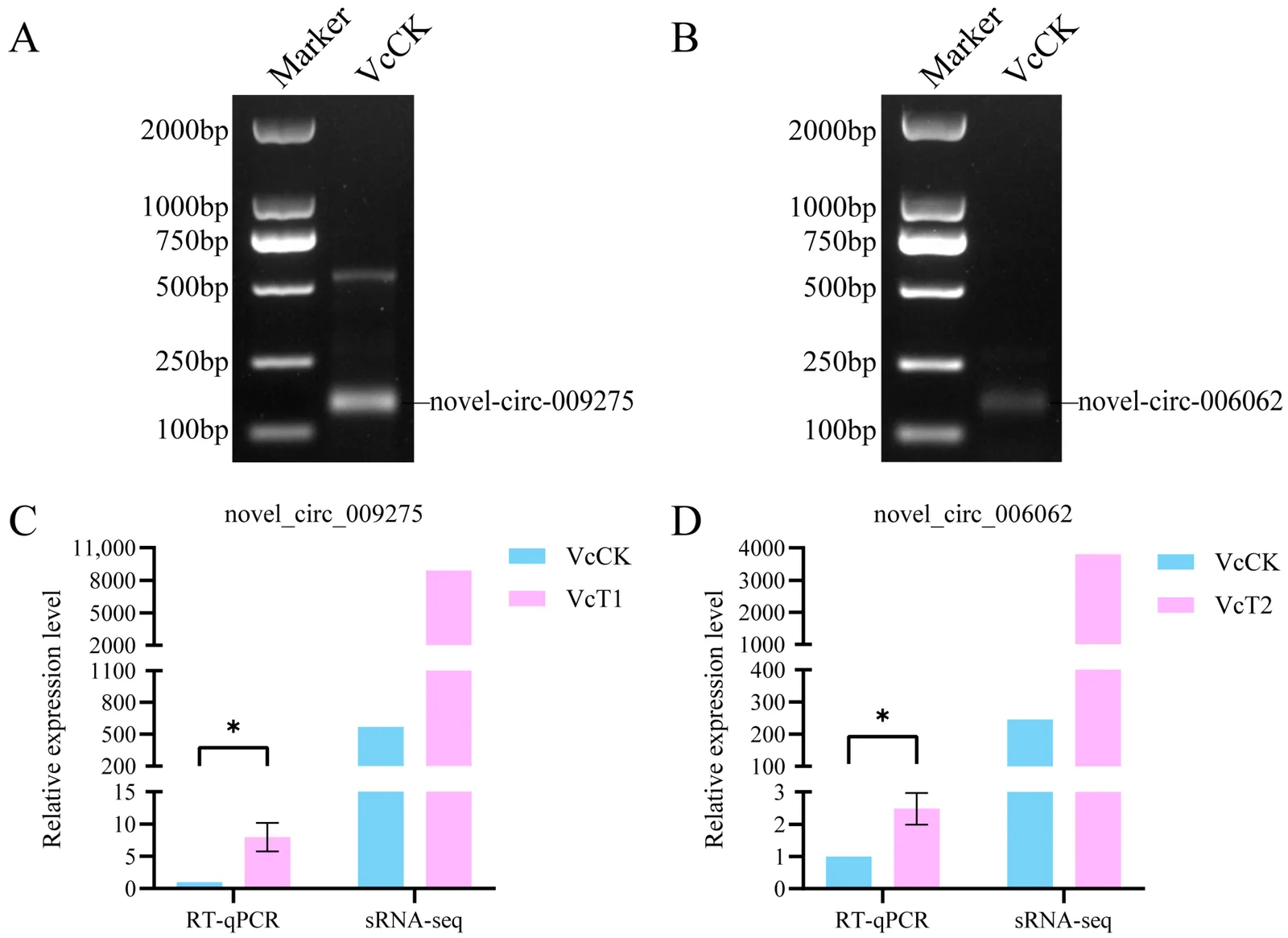
RT-PCR and RT-qPCR validation of novel-circ-009275 and novel-circ-006062 in *V. ceranae*. (**A**) Agarose gel electrophoresis for PCR amplification product, lane 1: DNA marker, lane 2: PCR product of novel-circ-009275 in the VcT1. (**B**) Agarose gel electrophoresis for PCR amplification product, lane 1: DNAmarker, lane 2: PCR product of novel-circ-006062 in the VcT2. (**C**) RT-qPCR result of novel-circ-009275 in *V. ceranae* infecting the midgut at 7 dpi (VcT1). (**D**) RT-qPCR result of novel-circ-006062 in *V. ceranae* infecting the midgut at 10 dpi (VcT2). Two-way ANOVA was applied according to the experimental design. Differences were considered statistically significant at *p* < 0.05. Statistical significance was defined as * (*p* < 0.05). VcCK: *V. ceranae* spores, VcT1 and VcT2: *V. ceranae* in the workers’ midguts at 7 days and 10 days post inoculation with spores.

Agarose gel electrophoresis showed that all two selected DEcircRNAs amplified specific bands consistent with the expected sizes, with no obvious non-specific amplification (Figure 6A,B).

RT-qPCR validation results showed that the expression trends of two randomly selected DEcircRNAs were consistent with those observed in the sequencing data, confirming the reliability of the sequencing data and the accuracy of the expression trends of the DEcircRNAs (Figure 6C,D).

## 4. Discussion

Circular RNAs (circRNAs) have emerged as a worldwide research hotspot in recent years. Nevertheless, relevant studies are predominantly limited to a small number of mammalian species including humans [30,31], while investigations into insect circRNAs started late and have advanced slowly. To date, global research on bee circRNAs has covered ovarian oviposition function [32], cerebral physiology [33], and intestinal development [34,35,36]. Accumulating evidence has demonstrated that circRNAs exert prominent immunomodulatory effects upon infection by pathogens such as bacteria and viruses [37,38,39].

The core objective of this current work was to characterize the dynamic profiling of endogenous circRNAs in *V. ceranae* during its infection of *A. c cerana*. Purified dormant spores (VcCK) were set as the baseline control to clarify the remodeling signatures of circular transcriptomes accompanying the transition from quiescent spores to intracellular parasitic stages. Through high-throughput sequencing and bioinformatic analyses, we observed a profound global remodeling of the circRNA expression landscape in microsporidia post-host infection, accompanied by a drastic overall reduction in circRNA abundance. The dominant circRNA subtype shifted from antisense circRNAs to single-exon circRNAs. Functional enrichment and ceRNA network prediction of screened DEcircRNAs implied their potential involvement in core biological processes of intracellular proliferation, including energy metabolism, protein processing, and stress adaptation.

Systematic elimination of potential technical confounders was performed to interpret the sharp decline in circRNA abundance. Identical protocols for total RNA extraction, linear RNA digestion with RNase R, library construction and sequencing were applied across all sample groups, with no disparities in experimental operations or bioinformatic pipelines among treatments. From a biological perspective, the dramatic drop in circRNA abundance tightly correlates with the life cycle transition of microsporidia. Dormant spores maintain a metabolically quiescent state with minimal basal biological activity. CircRNAs possess intrinsic structural advantages: lacking free termini, they resist ribonuclease degradation and exhibit extraordinary stability, rendering them ideal long-term stored regulatory molecules within spores to sustain post-transcriptional regulation during spore germination and early infection [40]. Following germination in the host midgut, microsporidia transit from metabolic dormancy to active proliferation, triggering genome-wide transcriptional reprogramming. Massive circRNAs pre-stored in spores are gradually degraded and cleared, leaving only a small subset associated with intracellular proliferation and stress tolerance, which ultimately accounts for the substantial reduction in total circRNA quantity. This hypothesis still lacks direct experimental evidence of circRNA degradation kinetics; further validations are required via simultaneous quantification of circRNA and linear mRNA turnover dynamics, as well as subcellular localization assays across distinct developmental stages.

In terms of structural characteristics, circRNAs derived from all three sample groups exhibited a concentrated length distribution ranging from 200~600 nt. The dynamic shift from antisense circRNA predominance in dormant spores to elevated proportions of single-exon circRNAs during intracellular parasitism is consistent with previous findings of stage-specific circRNA expression in Ascosphaera apis across mycelial and spore phases [41]. Functional prediction based on GO and KEGG enrichment of host genes of DEcircRNAs from each comparison group revealed participation in multiple pathways, including endoplasmic reticulum (ER) protein processing, endocytosis, ribosome biogenesis, and glycolysis/gluconeogenesis. Significant enrichment of the “RNA transport” pathway in the VcCK vs. VcT2 comparison implied that advancing infection drives *V. ceranae* to deploy sophisticated post-transcriptional regulatory machineries, modulating the expression of infection-related genes by governing nuclear export, subcellular localization and stability of mRNAs. As an obligate intracellular parasitic microsporidium, *V. ceranae* harbors a highly streamlined anabolic system, and the synthesis and folding of its virulence factors are largely dependent on the host ER [42]. Enrichment of the glycolysis/gluconeogenesis pathway aligns with existing consensus: intracellular pathogens tend to upregulate energy metabolism pathways to satisfy high energy demands during proliferation, which is logically congruent with the observation reported by Holt et al. [43] that microsporidian infection induces massive depletion of energy substrates in the bee midgut. Nevertheless, the aforementioned pathway enrichment results rely solely on bioinformatic annotation of host genes. Whether DEcircRNAs modulate the activity of these pathways via transcriptional or splicing regulation of their cognate host genes requires experimental validation using gene knockout or overexpression strategies.

ceRNAs constitute an emerging paradigm governing gene expression regulation, whose core mechanism lies in reciprocal modulation via competitive binding to shared miRNAs [44]. RNA transcripts harboring MREs, including lncRNAs, circRNAs, pseudogene transcripts and mRNAs, collectively form the ceRNA–miRNA–mRNA ternary regulatory axis. Here, we identified 62 and 64 DEcircRNAs with potential miRNA-binding capacity and downstream target mRNA regulatory roles in VcCK vs. VcT1 and VcCK vs. VcT2 groups, respectively. Among these, novel_circ_006653, novel_circ_006675 and novel_circ_006669 were each predicted to interact with five distinct miRNAs, displaying potent latent miRNA sponge activity. Functional enrichment of target mRNAs pointed to engagement in carbon metabolism, glycolysis/gluconeogenesis and ER protein processing. We therefore hypothesize that these DEcircRNAs competitively sequester miRNAs to relieve miRNA-mediated repression of target genes, thereby modulating the expression of host genes associated with energy metabolism and protein biosynthesis and constructing a permissive intracellular microenvironment for pathogen proliferation.

In summary, utilizing high-throughput sequencing and bioinformatic profiling, the present study preliminarily delineated profound shifts in circRNA expression signatures of *V. ceranae* throughout midgut infection, and predicted that subsets of DEcircRNAs mediate pathway regulation of energy metabolism and protein synthesis via ceRNA crosstalk. These findings offer novel insights into the molecular underpinnings of microsporidian–host interactions and provide valuable transcriptomic resources for screening candidate therapeutic targets against bee nosemosis. However, post-infection samples (VcT1 and VcT2) in this study were isolated from whole host midgut tissues; extracted *V. ceranae* RNAs are simultaneously influenced by two confounding variables: intrinsic pathogen developmental progression and dynamic shifts in the host midgut microenvironment. The current experimental design cannot quantitatively disentangle the independent contributions of these two factors, leaving an open question as to whether observed circRNA expression disparities arise from intrinsic developmental rhythms of the pathogen or passive remodeling imposed by the host niche. Follow-up sequencing of pathogens cultured in an in vitro intracellular-mimicking medium would help disentangle these confounding effects. Future research priorities include: molecular validation of circRNA–miRNA targeting interactions, single-cell profiling to dissect the respective contributions of pathogen development and host microenvironment, and functional verification via dual-luciferase reporter assays and other molecular experimental approaches.

## 5. Conclusions

**Figure 7:**
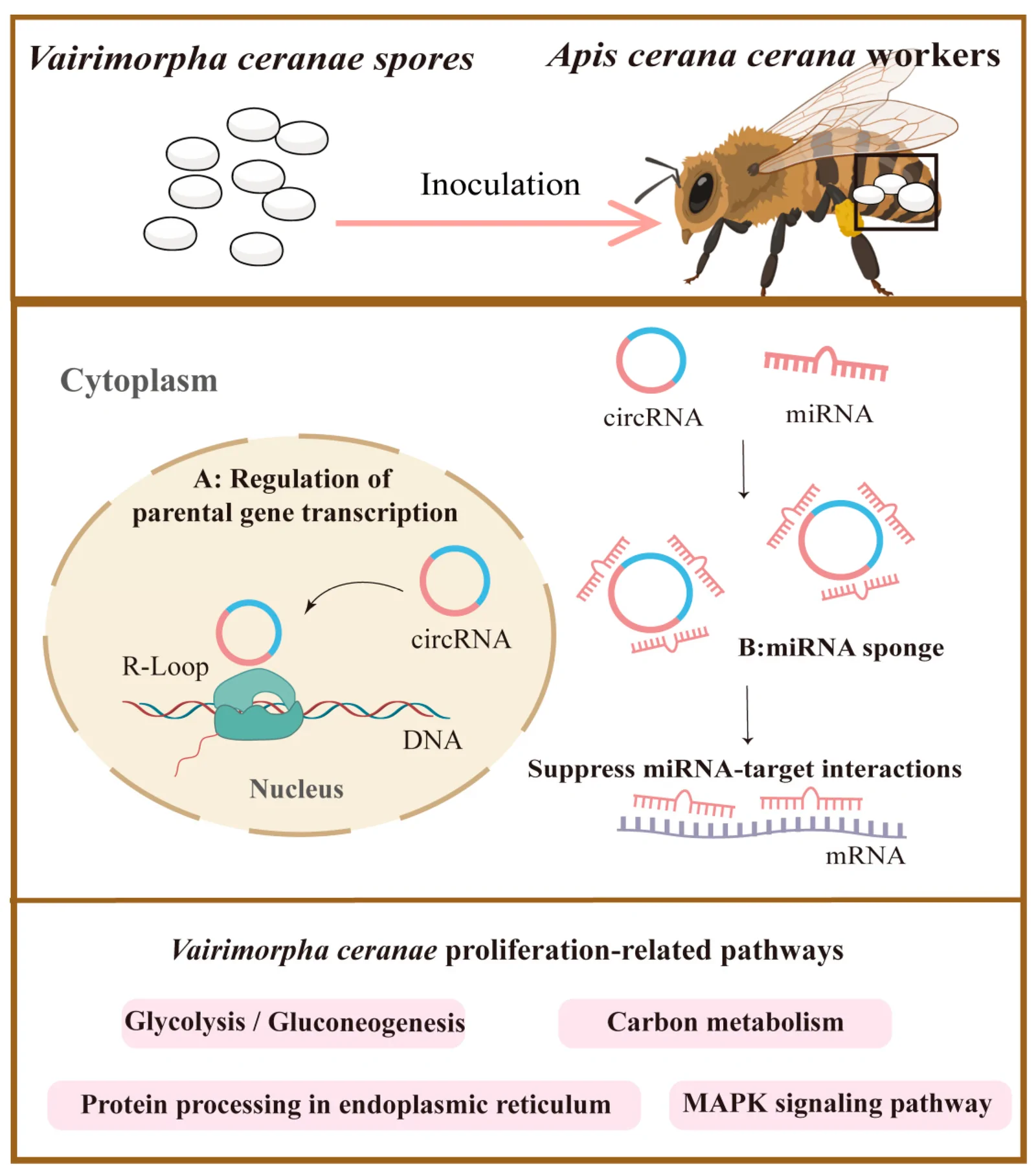
Regulatory model of circRNA expression and function during *V. ceranae* infection in *A. c. cerana* workers. VcCK: spores of Vairimorpha ceranae; VcT1 and VcT2: pathogen samples collected at 7 days and 10 days post-inoculation, respectively. Inoculation stage: Following inoculation, spores inject sporoplasm into host cells via the polar tube. Dynamic changes in expression profiles: Molecular functional mechanisms: (A) Regulation of parental gene transcription; (B) Acting as miRNA sponges (ceRNA mechanism) to suppress miRNA–target mRNA interactions. Downstream pathway regulation: circRNAs are involved in regulating glycolysis/gluconeogenesis, carbon metabolism, protein processing in endoplasmic reticulum, and the MAPK signaling pathway, thereby influencing the proliferation process of microsporidia.

In conclusion, this study characterizes the dynamic landscape of *V. ceranae* circRNAs during the infection of Asian honeybees, revealing that DEcircRNAs may serve as potential regulators of fungal intracellular proliferation and adaptation by modulating critical functional pathways such as carbon metabolism, glycolysis/gluconeogenesis, endoplasmic reticulum protein processing, and the MAPK signaling pathway via ceRNA networks. The proposed regulatory model (Figure 7) depicts a novel post-transcriptional regulatory layer underlying host–microsporidian interactions, and offers candidate molecular targets for the management of bee nosemosis.

## Figures and Tables

**Table 1:** Information of primers for RT-qPCR used in this work.

CircRNA ID	Sequence (5′-3′)
*actin*	F: GGTTGTTGATAGTGGAGATGG R: CACGACCAGCAATAGGAAT
novel_circ_009275	F: GCGTTATTTACCGCATCTGCT R: CACGTAGTTTCAATACAAGGTCTTC
novel_circ_006062	F: AAGGTCCATTTTCAAATTGTGTTTG R: ACCAGTGTGTACACCATGCAA

**Table 2:** Top 5 up- and down-regulated circRNAs in the VcCK vs. VcT1 comparison group.

CircRNA ID	Log2(Fold Change)	*p* Value	Source_Gene
novel_circ_002688	24.50	1.47 × 10^−9^	ncbi_9423198
novel_circ_002679	23.22	1.99 × 10^−6^	ncbi_9423198
novel_circ_002682	22.98	2.58 × 10^−5^	ncbi_9423198
novel_circ_002686	22.94	1.49 × 10^−7^	ncbi_9423198
novel_circ_004839	22.63	1.56 × 10^−3^	ncbi_9424396
novel_circ_005596	−24.88	2.69 × 10^−8^	NA
novel_circ_005553	−24.82	3.19 × 10^−8^	NA
novel_circ_005597	−24.74	3.97 × 10^−8^	NA
novel_circ_005552	−24.73	4.12 × 10^−8^	NA
novel_circ_007900	−23.16	5.60 × 10^−6^	ncbi_9424317

NA: Not available; indicates that no source gene could be annotated for the corresponding circRNA.

**Table 3:** Top 5 up- and down-regulated circRNAs in the VcCK vs. VcT2 comparison group.

CircRNA ID	Log2(Fold Change)	*p* Value	Source_Gene
novel_circ_002398	25.07	7.90 × 10^−4^	ncbi_9422726
novel_circ_009047	24.87	8.25 × 10^−4^	ncbi_9423860
novel_circ_003865	24.79	8.40 × 10^−4^	ncbi_9424586
novel_circ_002392	24.78	4.47 × 10^−4^	ncbi_9422729
novel_circ_005990	24.53	8.93 × 10^−4^	ncbi_9424024
novel_circ_005596	−24.88	2.50 × 10^−9^	NA
novel_circ_005553	−24.82	2.98 × 10^−9^	NA
novel_circ_005597	−24.75	3.75 × 10^−9^	NA
novel_circ_005552	−24.73	3.91 × 10^−9^	NA
novel_circ_007900	−23.16	8.62 × 10^−7^	ncbi_9424317

NA: Not available; indicates that no source gene could be annotated for the corresponding circRNA.

## Data Availability

Data will be made available on request.
